# Editorial: Mitochondria in Skeletal Muscle Health, Aging and Diseases

**DOI:** 10.3389/fphys.2016.00446

**Published:** 2016-10-06

**Authors:** Gilles Gouspillou, Russell T. Hepple

**Affiliations:** ^1^Département des Sciences de l'Activité Physique, Faculté des Sciences, Université du Québec à MontréalMontreal, QC, Canada; ^2^Groupe de Recherche en Activité Physique AdaptéeMontreal, QC, Canada; ^3^Centre de Recherche de l'Institut, Universitaire de Gériatrie de MontréalMontreal, QC, Canada; ^4^Department of Kinesiology and Physical Education, McGill UniversityMontreal, QC, Canada; ^5^Meakins-Christie Laboratory, Research Institute of the McGill University Health CentreMontreal, QC, Canada

**Keywords:** muscle, aging, mitochondria, metabolism, mitophagy, mitochondrial dynamics, bioenergetics, muscle fibers

Mitochondria are fascinating organelles regulating many critical cellular processes for skeletal muscle physiology. Indeed, they play central roles in muscle cell metabolism, energy supply, the regulation of energy-sensitive signaling pathways, reactive oxygen species (ROS) production/signaling, calcium homeostasis and the regulation of apoptosis (Brookes et al., 2004). Given these multifaceted roles of mitochondria in fundamental aspects of skeletal muscle cell physiology, it is not surprising that mitochondrial dysfunction has been implicated in a large number of adverse conditions affecting skeletal muscle health. This includes for instance the aging-related loss of muscle mass and function (Dirks and Leeuwenburgh, 2004; Short et al., 2005; Chabi et al., 2008; Gouspillou et al., 2010, 2014a,b; Picard et al., 2010; Hepple, 2014), disuse-induced muscle atrophy (Min et al., 2011), ventilator-induced diaphragmatic dysfunction (Picard et al., 2015), Duchenne and collagen muscular dystrophies (Godin et al., 2012; Bernardi and Bonaldo, 2013), long-term muscle dysfunction induced by chemotherapy treatment (Gouspillou et al., 2015; Power et al., 2016), and the development of insulin resistance (Goodpaster, 2013).

While the importance of normal mitochondrial function is well recognized for muscle physiology, there are important aspects of mitochondrial biology that are still poorly understood/investigated in the highly specialized muscle tissue. These include mitochondrial dynamics (fusion and fission processes), morphology and processes involved in mitochondrial quality control (mitophagy). Defining the mechanisms regulating these different aspects of mitochondrial biology, their importance for muscle physiology, as well as the interrelations existing between mitochondrial function, morphology, dynamics and mitophagy will be critical to further increase our understanding of the role played by mitochondria in skeletal muscle physiology and pathophysiology. The aim of the present research topic was therefore to bring together key experiments, advances, knowledge and new findings related to all aspects of mitochondrial biology in healthy and/or diseased muscle cells.

The accurate assessment of mitochondrial function is of crucial importance to dissect the role played by mitochondrial dysfunction in pathologies affecting skeletal muscles. It is also essential for the clear identification of mitochondrial adaptations to various interventions, such as nutritional or physical activity interventions. In the present research topic, Conley et al. provide a promising approach to assess mitochondrial oxidative phosphorylation *in vivo* by monitoring changes in mitochondrial NAD(P)H using ^31^P NMR spectroscopy. In their study, Conley et al. show that a unique resonance (−11.05 ppm) in the *in vivo* ^31^P spectrum provides a natural indicator of mitochondrial oxidation that is sensitive to the increase in mitochondrial phosphorylation rate induced by exercise training in elderly individuals.

One of the commonly used methods to assess mitochondrial function *in vitro* in skeletal muscle is the preparation of permeabilized myofibers (Kuznetsov et al., 2008). To be applied in humans, this technique requires biopsies to collect muscle samples. While the Bergstrom biopsy technique (Bergstrom, 1975) is the gold standard to collect skeletal muscle samples, a growing interest now surrounds microbiospsies due to their lower level of invasiveness (Hayot et al., 2005). However, no previous in-depth validation of this method for the assessment of mitochondrial function in permeabilized myofibers and for the assessment of muscle phenotype had been performed to date. Hughes et al. therefore compared mitochondrial bioenergetics in muscle sample obtained through Bergstrom biopsies vs. microbiopsies. They reveal that microbiopsies can provide a reliable assessment of mitochondrial bioenergetics only when assay conditions are supplemented with the myosin ATPase inhibitor Blebistatin. However, the authors highlight that caution should be taken when assessing muscle fiber type composition using the microbiopsy approach, since significant differences in fiber type proportion were observed between the two approaches (Hughes et al.).

Oxidative stress is thought to play an important role in skeletal muscle dysfunction and atrophy seen in aging, disuse, and many skeletal muscle pathologies (Powers et al., 2012; Johnson et al., 2013). Because they are considered as one of the main sources of ROS production, mitochondria are a key focus in the field of oxidative stress. While reactive oxygen and nitrogen species were initially only seen as detrimental for muscle cells, it is now recognized that these reactive species are essential for normal skeletal muscle physiology (Sohal and Orr, 2012), mainly through the reversible redox post-translational modifications they can induce. The ability to accurately quantify reversible redox post-translational modifications is therefore critical to investigate the mechanisms by which mitochondrial oxidative stress contributes to skeletal muscle dysfunction in diseases. In their article, Kramer et al. provide a detailed review of the available literature on reversible redox post-translational modifications and mitochondrial and skeletal muscle function. They then provide critical review on current approaches to assess reversible redox post-translational modifications (Kramer et al.).

Several studies have implicated altered kinetic properties of the adenine nucleotide translocator (ANT) in the aging-related impairment in mitochondrial energetics in skeletal muscle cells (Yan and Sohal, 1998; Gouspillou et al., 2014b). In the present research topic, Diolez et al. formulate the interesting hypothesis that these alterations in ANT could represent a protective mechanism to limit ROS production in aged muscle mitochondria while moderately disrupting mitochondrial energetics. Considering the importance of ROS as therapeutic targets, this hypothetical mechanism deserves further study.

The present research topic also provides readers with fundamental advancement in our understanding of the regulation of mitochondrial function in skeletal muscle cells. Indeed, in an elegant study, Lark et al. provide evidence that Protein Kinase A (PKA) can regulate mitochondrial energetics and H_2_O_2_ emission. Using PKA inhibitors and various mitochondrial substrates, they show that this regulation occurs at the level of Complex I. Finally, they provide new insights on how mitochondrial cyclic adenosine monophosphate (cAMP) production, cAMP being a positive regulator of PKA, is regulated (Lark et al.).

Understanding how nutrition modulates mitochondrial biology in muscle cells is of tremendous importance in the field of medicine. For instance, mitochondrial dysfunction has been suggested to be causally involved in obesity-induced insulin resistance and in the pathophysiology of type II diabetes (Goodpaster, 2013). Precisely defining how skeletal muscle mitochondria respond to obesogenic diet feeding is therefore of critical importance. In the present research topic, Putti et al. provide readers with a mini-review focused on the impacts of different dietary fat sources on mitochondrial bioenergetics, morphology and dynamics in skeletal muscle cells in the context of insulin-resistance. They also highlight the pressing need for mechanistic studies to confirm *in vivo* the relationship between mitochondrial morphology and dynamics and the development of insulin-resistance (Putti et al.).

Besides being of particular interest for the field of medicine, defining the impact of nutrition on mitochondrial biology is also an important research topic in the field of exercise physiology. The present research topic features two important review articles in this field. The first one, written by Craig et al. critically reviews the available literature on the utilization of small nutrients, such as caffeine, green tea extracts, polyphenols and amino-acids to enhance the impact of exercise training on mitochondrial biogenesis. They also provide recommendations and guidance for future studies that are required to explore the efficacy of these nutrients in humans, as well as the exercise setting in which they may prove beneficial (Craig et al.). The second review, written by Affourtit et al., first provides a state-of-the-art review of the available literature on the beneficial effects of dietary nitrate on human performance. Affourtit et al. then critically review the available experimental data in relation to the underlying mechanisms, with a particular emphasis on the impact of nitrate on mitochondrial bioenergetics.

It is now well established that low birth weight is associated with an increase in the risk of developing disease in later life such as coronary heart disease, diabetes, hypertension and stroke (see de Boo and Harding, 2006; Gluckman et al., 2008 for detailed reviews). Maternal undernutrition ranks as one of the most important causes that can lead to low birth weight (de Boo and Harding, 2006). In the present research topic, Beauchamp and Harper review the available evidence indicating that *in utero* undernutrition results in a metabolic reprogramming in both cardiac and skeletal muscles, characterized for instance by a reduction in mitochondrial content and respiration in the offspring. They also provide insights into the underlying mechanisms and provide a rationale linking the metabolic alterations resulting from *in utero* undernutrition to the increased risk of developing metabolic diseases (Beauchamp and Harper).

As mentioned earlier, mitochondrial dysfunction has been implicated in many conditions associated with muscle atrophy, including aging, cancer cachexia, and disuse-induced muscle atrophy (Dirks and Leeuwenburgh, 2004; Short et al., 2005; Chabi et al., 2008; Gouspillou et al., 2010, 2014a,b; Picard et al., 2010, 2015; Min et al., 2011; Hepple, 2014; Argilés et al., 2015). While the field to date has mainly focused on mitochondrial function (i.e., energetics, ROS production and mitochondrial mediated apoptosis), a growing interest now surrounds mechanisms involved in mitochondrial quality control (i.e., mechanisms responsible for the degradation of damaged / dysfunctional mitochondria). In the present research topic, Romanello and Sandri provide readers with a thorough and critical review on the current knowledge linking mitochondrial function, dynamics and quality control in the regulation of muscle mass. They also highlight several research avenues and challenges that will undoubtedly stimulate the field (Romanello and Sandri).

In the present research topic, Ryan et al. contributed a very interesting review paper focused on skeletal muscle and endothelial cell mitochondria in the setting of Critical Limb Ischemia. The latter is the most severe clinical presentation of peripheral arterial disease and manifests as chronic ischaemic “rest pain” and/or ischaemic skin lesions (Minar, 2009). Unfortunately, there is no effective way to treat the muscle myopathies caused by this disease. In their review, Ryan et al. first highlight the importance of skeletal muscle in the manifestation of Critical Limb Ischemia, and then provide a strong and exciting rationale for a role for endothelial cell and skeletal muscle mitochondria in the patient outcomes. As such, they propose that limb muscle and endothelial cell mitochondria should be considered as targets for novel therapeutic intervention (Ryan et al.).

Skeletal muscles have an impressive capacity to adapt to mechanical and physiological challenges by changing their phenotype in terms of size, fiber type, capillarization levels and aerobic capacity. Although skeletal muscle plasticity has been the focus of intense research effort, the molecular mechanisms underlying muscle plasticity are still incompletely understood. Since the seminal paper published by Spiegleman's group in 2002 (Lin et al., 2002), in which PGC-1α over-expression was shown to result in an increase in the proportion of slow-oxidative fibers, PGC-1α has attracted sustained attention. In the present research topic, Kupr and Handschin discuss recent advances in our understanding of how PGC-1α regulates skeletal muscle cell plasticity in health and disease. They also highlight further avenues of research to fully decrypt how PGC-1α influences muscle plasticity in health and disease (Kupr and Handschin).

Although often overlooked, our endocrine system exerts an important control on the regulation of mitochondrial mass and function in skeletal muscle cells, especially through thyroid hormones (Salvatore et al., 2014). Amongst thyroid hormones exerting control on mitochondrial biology in skeletal muscle cells, 3,5,3′-Triiodo-L-thyronine (T3) has been extensively studied (Salvatore et al., 2014). In the present research topic, Lumbardi et al. review the experimental evidence indicating that 3,5-diiodo-L-thyronine (T2), and emerging iodothyronines also impact mitochondrial metabolism in skeletal muscle cells (Lombardi et al.). These novel aspects of thyroid physiology reviewed in Lombardi et al. open new perspectives for understanding the involvement of skeletal muscle mitochondria in systemic consequences of hypo- and hyper-thyroidism.

Because of the diversity and the quality of the articles compiled herein, we feel the present research topic was a success. We hope that it will not only provide readers with new insights and viewpoints on the role played by mitochondria in skeletal muscle health, aging and diseases, but will also serve as a platform to stimulate new ideas, experiments and research projects for further advances in the field.

## Author contributions

GG and RH co-wrote and approved this Editorial.

### Conflict of interest statement

The authors declare that the research was conducted in the absence of any commercial or financial relationships that could be construed as a potential conflict of interest.
